# Investigating the Thermal Transformations of Chlorogenic Acids During Dry‐Heating Processing of *Lonicerae Japonicae Flos*

**DOI:** 10.1155/ijfo/7566064

**Published:** 2026-05-11

**Authors:** Peishan Zhao, Kaixiang Dong, Yan Liu, Hongyang Zhang, Qiangqiang Jia, Yuerong Wang, Ping Hu, Min Zhang

**Affiliations:** ^1^ Shanghai Frontiers Science Center of Optogenetic Techniques for Cell Metabolism, School of Pharmacy, East China University of Science and Technology, Shanghai, China, ecust.edu.cn; ^2^ Department of Pharmaceutical Engineering, School of Pharmacy, East China University of Science and Technology, Shanghai, China, ecust.edu.cn; ^3^ Shanghai Key Laboratory of Functional Materials Chemistry, School of Chemistry and Molecular Engineering, East China University of Science and Technology, Shanghai, China, ecust.edu.cn; ^4^ The College of Pharmacy, Qinghai University, Xining, China, qhu.edu.cn

**Keywords:** honeysuckle, isochlorogenic acid, kinetic transformation, thermal degradation, thermodynamic transformation

## Abstract

*Lonicerae japonicae Flos* (*LjF*, honeysuckle) is a major component of beverages and healthcare products, as well as a key botanical resource in traditional Chinese medicine. Chlorogenic acids (CGAs) are the main bioactive components of *LjF*, possessing antioxidant, anti‐inflammatory, antiviral, and other biological activities, among which CGA serves as the key marker compound for *LjF* quality evaluation. In the present work, *LjF* samples were subjected to controlled dry heating and analyzed by LC‐DAD and UPLC‐QTOF‐MS. Progressive degradation of neochlorogenic acid, cryptochlorogenic acid, and isochlorogenic acid A was accompanied by concomitant accumulation of CGA, isochlorogenic acid B, and isochlorogenic acid C. Furthermore, the effects of temperature and time on thermal transformation, along with key chemical markers for different heat treatment conditions were clarified via principal component analysis of chemical changes. Mechanistic investigations revealed that isochlorogenic acid A undergoes competitive cleavage of the C‐3 or C‐5 caffeoyl ester bond to yield neochlorogenic acid (kinetic product) or CGA (thermodynamic product), followed by intramolecular acyl migration to form isochlorogenic acid B and isochlorogenic acid C, with isochlorogenic acid C identified as the most stable dicaffeoyl isomer. The interconversion of monocaffeoyl isomers leads to a dynamic equilibrium, with CGA being the predominant and most stable form. These results provide the comprehensive thermal‐transformation map for CGAs in *LjF* and offer practical guidance for optimizing drying, roasting, and extraction protocols in the development of high‐quality *LjF*, an edible and medicinal homologous resource.

## 1. Introduction

*Lonicerae japonicae Flos* (*LjF*, Jinyinhua, Honeysuckle) originates from the dried flower buds or partially opened flowers of *Lonicera japonica* Thunb, belonging to the Caprifoliaceae family. It is primarily distributed in East Asian countries including China, South Korea, and Japan. First introduced to the United States [1] in the 19th century, it has since been reported in Europe, Canada [2], and other areas. Presently, the applications of *LjF* extend significantly beyond medicinal purposes, finding extensive development within commercial sectors including food, beverages, and healthcare products. With its broad application prospects, its annual demand in China reaches approximately 30,000 t. Existing studies on compositional changes are primarily confined to the biological field [3] and to storage or drying methods [4]. Research on the transformation of chlorogenic acids (CGAs), the key bioactive constituents of *LjF* during dry heating is currently lacking.

**Figure 1:**
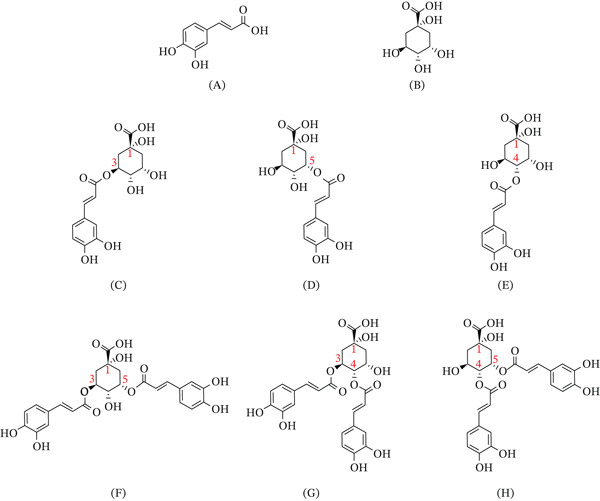
Chemical structures of CGAs. (A) caffeic acid; (B) quinic acid; (C) chlorogenic acid (3‐O‐caffeoylquinic acid); (D) neochlorogenic acid (5‐O‐caffeoylquinic acid); (E) cryptochlorogenic acid (4‐O‐caffeoylquinic acid); (F) isochlorogenic acid A (3,5‐di‐O‐caffeoylquinic acid); (G) isochlorogenic acid B (3,4‐di‐O‐caffeoylquinic acid); and (H) isochlorogenic acid C (4,5‐di‐O‐caffeoylquinic acid).

In *LjF*, CGAs primarily comprise six specific isomers: neochlorogenic acid, CGA, cryptochlorogenic acid, isochlorogenic acid A, isochlorogenic acid B, and isochlorogenic acid C [5], whose chemical structures are depicted in Figure 1. Neochlorogenic acid, CGA, and cryptochlorogenic acid are termed monocaffeoylquinic acids; simultaneously, isochlorogenic acid A, isochlorogenic acid B, and isochlorogenic acid C are classified as dicaffeoylquinic acids.

As the key marker compound for evaluating the quality of *LjF* [6], CGA exhibits a broad spectrum of pharmacological activities, including anti‐inflammatory [7], antibacterial [8], antioxidant [9], antiviral [10], hepatoprotective effect [11], cardioprotective effect [12], antiobesity action [13], amelioration of ovarian impairment [14], cancer prevention and treatment [15], and reduction of the risk of neurological diseases such as Alzheimer′s disease [16], cognitive dysfunction [17], and Parkinson′s disease [18].

Likewise, although dicaffeoylquinic acids have not been studied extensively as CGA to date, they still exhibit a broad spectrum of pharmacological activities [19]. Notably, isochlorogenic acid C, among the dicaffeoylquinic acids, exhibits the most potent antioxidant and antibacterial activities, whereas isochlorogenic acid A and isochlorogenic acid B demonstrate comparable potency [20, 21]. However, isolation, purification, identification, and quality evaluation of isochlorogenic acids B and C predominantly rely on extraction from natural sources [22–24], resulting in prohibitively high costs that impede related research.

Current investigations into the thermal stability of monocaffeoylquinic acids are relatively comprehensive [25], yet predominantly focus on CGA itself under ultrahigh temperatures [26] or in solution [27]. Most existing research employed either temperature or time as a single variable, centered on CGA; however, the exploration of its transformation and interrelationship with dicaffeoylquinic acids was insufficient.

In this contribution, via dry‐heat methodology with solvent effects excluded, the coupled effects of heating temperature and duration on active components, CGAs, in *LjF* were systematically investigated, aiming to enhance the contents of CGA, isochlorogenic acid B, and isochlorogenic acid C. Furthermore, the thermal transformation relationships of CGAs in *LjF* were systematically elaborated at the mechanistic level, along with a novel mechanistic insight: neochlorogenic acid and CGA are the kinetic and thermodynamic degradation products of isochlorogenic acid A, respectively.

## 2. Materials and Methods

### 2.1. Materials and Reagents

*LjF* samples originated from Henan Province, China. HPLC grade methanol was procured from Shanghai Wohua Chemical Co. Ltd (Shanghai, China). MS grade acetonitrile was purchased from Merck (United States). Formic acid was sourced from Shanghai Lingfeng Chemical Reagent Co. Ltd (Shanghai, China). The CGA reference standard (≥ 98% pure) was purchased from Shanghai Dibai Biotechnology Co. Ltd (Shanghai, China). The reference standards for isochlorogenic acid A (≥ 98% pure), isochlorogenic acid B (≥ 98% pure), and isochlorogenic acid C (≥ 98% pure) were obtained from Chengdu Yijierui Biotechnology Co. Ltd (Chengdu, China). The ultrapure water was prepared using a PLUS‐E2‐10TJ laboratory ultrapure water purification system (Nanjing Eped Technology Development Co. Ltd, Nanjing, China). The DZX‐3 (6020B) vacuum drying oven (Shanghai Fuma Experimental Equipment Co. Ltd.) was used for the dry‐heat treatment process.

### 2.2. Probing the Transformation of CGAs

#### 2.2.1. Sample Preparation

A total of 5 g of *LjF* powder samples were placed in the vacuum drying oven and heated at 60°C, 80°C, 100°C, 120°C, 140°C, and 160°C, respectively, for 15 min. Separate batches of the *LjF* powder samples were heated in the oven at 80°C, 100°C, and 120°C for 30, 60, 90, and 120 min, respectively. Following heat treatment, 0.1 g of each sample was extracted with 10 mL of 80% (V/V) methanol with an ultrasonic extractor (power 300 W, frequency 40 kHz) for 60 min. The samples were then filtered through a 0.22‐*μ*m nylon filter membrane and subjected to analysis.

#### 2.2.2. HPLC Analysis Method

Chromatographic analysis was performed using an Agilent 1200 HPLC system (Agilent Technologies, China), equipped with a thermostatted compartment, a UV diode array detector (DAD), a binary pump, and an autosampler. Separation was achieved on a WonCract ODS‐2 column (4.6 × 150 mm, 5 *μ*m) maintained at 30°C. The mobile phase consisted of Solvent A (0.1% formic acid, V/V) and Solvent B (acetonitrile), employing the following gradient program: 0–8 min, 14%–19% B; 8–14 min, 19% B; 14–34 min, 19%–31% B; 34–35 min, 31%–90% B; 35–40 min, 90% B; 40–42 min, 90%–14% B; 42–50 min, 14% B. The flow rate was 0.7 mL/min, the injection volume was 10 *μ*L, and UV detection was performed at 327 nm. The method validation of HPLC in this study, covering accuracy, stability, repeatability, and recovery, is included in Supporting Information 1

#### 2.2.3. LC‐QTOF‐MS Analysis Method

Analysis was conducted using an Agilent 1290 UHPLC system (Agilent Technologies, China), equipped with a thermostatted compartment, a UV DAD, a binary pump, and an autosampler. The chromatographic conditions were adopted from Section 2.2.2. The system was equipped with the same WonCr act ODS‐2 column (4.6 × 150 mm, 5 *μ*m) as used in Section 2.2.2, because UHPLC systems are compatible with conventional HPLC columns. Moreover, a splitter was installed between the LC and the MS to regulate the eluent flow rate to match the mass spectrometer requirements. For mass spectrometric analysis, MS data were acquired in TOF mode using ESI (−) negative ion mode scan. The operating parameters were configured as follows: drying gas and collision gas, N_2_; drying gas temperature, 350°C; drying gas flow rate, 10 L/min; nebulizer pressure, 30 psi; capillary voltage, 3000 V; skimmer voltage, 65 V; octabar RF voltage, 750 V; capillary exit voltage, 150 V; and mass scan range, *m/z* 50–1000. For targeted MS/MS analysis, collision energy values were individually set at 20 and 40 eV based on the fragmentation characteristics of the target ions.

#### 2.2.4. Kinetic and Thermodynamic Transformation Process of CGAs

A total of 5 g of *LjF* powder samples were placed in the vacuum drying oven and heated at 120°C for 5 and 120 min, respectively. Following heat treatment, the samples were rapidly cooled. Subsequently, 0.1 g of both heat‐treated samples alongside the untreated sample were individually immersed in 10 mL of 80% (V/V) methanol. Extraction was performed using ice‐bath ultrasonic extraction (power 300 W, frequency 40 kHz) for 10 min. The resulting extracts were filtered and then subjected to analysis. The HPLC method employed was identical to that described in Section 2.2.2.

## 3. Results and Discussion

### 3.1. Identification of the CGAs in *LjF*

#### 3.1.1. Identification of the CGAs by HPLC

**Figure 2:**
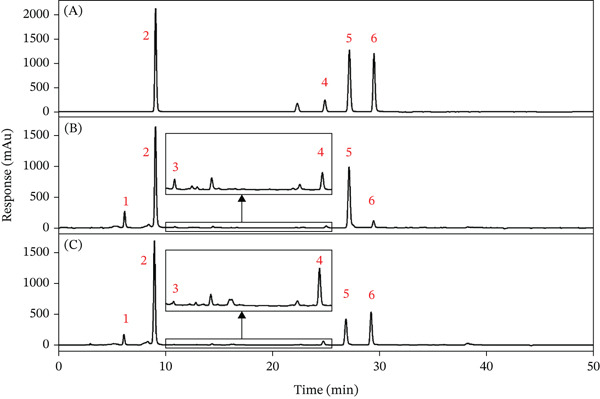
HPLC chromatograms of the typical samples. (A) HPLC chromatogram of the reference standard; (B) HPLC chromatogram of the untreated sample; and (C) HPLC chromatogram of the sample treated at 120°C for 120 min. Peak 1, neochlorogenic acid; Peak 2, chlorogenic acid; Peak 3, cryptochlorogenic acid; Peak 4, isochlorogenic acid B; Peak 5, isochlorogenic acid A; and Peak 6, isochlorogenic acid C.

Figure 2 depicts the chromatograms of the reference standards, the untreated *LjF* sample, and the sample subjected to heat treatment at 120°C for 120 min, under the HPLC conditions specified in Section 2.2.2.

#### 3.1.2. Identification of the CGAs by LC‐QTOF‐MS

**Figure 3:**
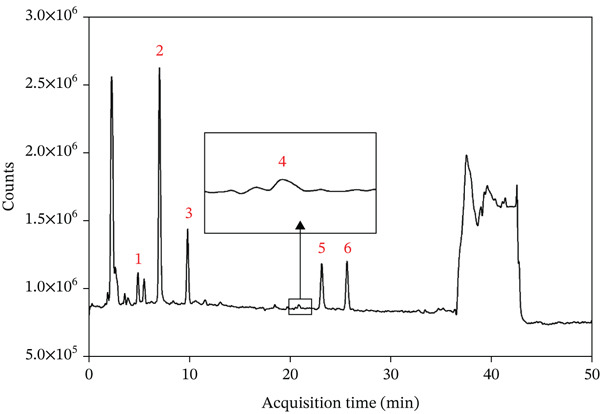
Total ion chromatogram (TIC) of the sample treated at 140°C for 15 min under the conditions specified in Section 2.2.4. Peak 1, neochlorogenic acid; Peak 2, chlorogenic acid; Peak 3, cryptochlorogenic acid; Peak 4, isochlorogenic acid B; Peak 5, isochlorogenic acid A; and Peak 6, isochlorogenic acid C.

Figure 3 expresses the chromatograms obtained from total ion chromatogram (TIC) of the sample treated at 140°C for 15 min under the LC‐QTOF‐MS conditions detailed in Section 2.2.3.

Based on the mass spectrometric data acquired in this work and previous works [28–31], Peak 1 was identified as neochlorogenic acid, Peak 2 as CGA, Peak 3 as cryptochlorogenic acid, Peak 4 as isochlorogenic acid B, Peak 5 as isochlorogenic acid A, and Peak 6 as isochlorogenic acid C.

Since LC‐QTOF‐MS was used only for qualitative identification, full method validation was not performed. However, to ensure reliable identification, the mass accuracy was maintained within 5 ppm, and the retention time repeatability was RSD < 0.55% by analyzing 12 samples. The fragment ions were stable across repeated injections. The MS/MS data for the six principal analytes targeted in this study are presented in Table 1. The six most abundant fragment ions were selected and sorted in descending order of abundance.

**Table 1:** MS/MS data from the LC‐QTOF‐MS spectra of the samples after heat treatment.

Types	tR (min)	Formula	[M−H]^−^	MS/MS (20 eV)	MS/MS (40 eV)	Analytes
Monocaffeoylquinic acids	5.034	C_16_H_18_O_9_	353.0854	191.0532 [M−H−C_9_H_6_O_3_]^−^, 179.0324 [M−H−C_7_H_10_O_5_]^−^, 135.0425 [M−H−C_7_H_10_O_5_−CO_2_]^−^, 161.0244 [M−H−C_7_H_12_O_6_]^−^, 173.0440 [M−H−C_9_H_6_O_3_−H_2_O]^−^, 155.0350 [M−H−C_9_H_6_O_3_−2H_2_O]^−^	135.0426 [M−H−C_7_H_10_O_5_−CO_2_]^−^, 191.0525 [M−H−C_9_H_6_O_3_]^−^, 179.0307 [M−H−C_7_H_10_O_5_]^−^, 161.0212 [M−H−C_7_H_12_O_6_]^−^, 127.0359 [M−H−C_9_H_6_O_3_−H_2_O−CO_2_]^−^, 143.0312 [M−H−C_7_H_12_O_6_−H_2_O]^−^	Neochlorogenic acid
	7.164	C_16_H_18_O_9_	353.0863	191.0532 [M−H−C_9_H_6_O_3_]^−^, 173.0424 [M−H−C_9_H_6_O_3_−H_2_O]^−^, 161.0213 [M−H−C_7_H_12_O_6_]^−^, 135.0423 [M−H−C_7_H_10_O_5_−CO_2_]^−^, 127.0350 [M−H−C_9_H_6_O_3_−H_2_O−CO_2_]^−^, 155.0324 [M−H−C_9_H_6_O_3_−2H_2_O]^−^	191.0533 [M−H−C_9_H_6_O_3_]^−^, 127.0376 [M−H−C_9_H_6_O_3_−H_2_O−CO_2_]^−^, 135.0426 [M−H−C_7_H_10_O_5_−CO_2_]^−^, 173.0415 [M−H−C_9_H_6_O_3_−H_2_O]^−^, 161.0198 [M−H−C_7_H_12_O_6_]^−^, 155.0338 [M−H−C_9_H_6_O_3_−2H_2_O]^−^	Chlorogenic acid
	8.620	C_16_H_18_O_9_	353.0854	191.0539 [M−H−C_9_H_6_O_3_]^−^, 179.0353 [M−H−C_7_H_10_O_5_]^−^, 173.0225 [M−H−C_9_H_6_O_3_−H_2_O]^−^, 133.5423 [M−H−C_7_H_10_O_5_−HCOOH]^−^, 135.0403 [M−H−C_7_H_10_O_5_−CO_2_]^−^, 161.0107 [M−H−C_7_H_12_O_6_]^−^	191.0500 [M−H−C_9_H_6_O_3_]^−^, 127.0376 [M−H−C_9_H_6_O_3_−H_2_O−CO_2_]^−^, 161.0426 [M−H−C_7_H_12_O_6_]^−^, 143.0223 [M−H−C_7_H_12_O_6_−H_2_O]^−^, 173.0303 [M−H−C_9_H_6_O_3_−H_2_O]^−^, 179.9512 [M−H−C_7_H_10_O_5_]^−^	Cryptochlorogenic acid
Dicaffeoylquinic acids	20.900	C_25_H_24_O_12_	515.1174	353.0886 [M−H−C_9_H_6_O_3_]^−^, 179.0302 [M−H−C_9_H_6_O_3_−C_7_H_10_O_5_]^−^, 173.0442 [M−H−2C_9_H_6_O_3_−H_2_O]^−^, 191.0526 [M−H−2C_9_H_6_O_3_]^−^, 335.0712 [M−H−C_9_H_6_O_3_−H_2_O]^−^, 135.0440 [M−H−C_9_H_6_O_3_−C_7_H_10_O_5_−CO_2_]^−^	179.0326 [M−H−C_9_H_6_O_3_−C_7_H_10_O_5_]^−^, 173.0422 [M−H−2C_9_H_6_O_3_−H_2_O]^−^, 135.0443 [M−H−C_9_H_6_O_3_−C_7_H_10_O_5_−CO_2_]^−^, 191.0521 [M−H−2C_9_H_6_O_3_]^−^, 161.0250 [M−H−C_9_H_6_O_3_−C_7_H_12_O_6_]^−^, 155.0308 [M−H−2C_9_H_6_O_3_−2H_2_O]^−^	Isochlorogenic acid B
	23.171	C_25_H_24_O_12_	515.1161	353.0858 [M−H−C_9_H_6_O_3_]^−^, 191.0537 [M−H−2C_9_H_6_O_3_]^−^, 179.0324 [M−H−C_9_H_6_O_3_−C_7_H_10_O_5_]^−^, 135.0427 [M−H−C_9_H_6_O_3_−C_7_H_10_O_5_−CO_2_]^−^, 161.0235 [M−H−C_9_H_6_O_3_−C_7_H_12_O_6_]^−^, 173.0447 [M−H−2C_9_H_6_O_3_−H_2_O]^−^	191.0540 [M−H−2C_9_H_6_O_3_]^−^, 179.0328 [M−H−C_9_H_6_O_3_−C_7_H_10_O_5_]^−^, 135.0429 [M−H−C_9_H_6_O_3_−C_7_H_10_O_5_−CO_2_]^−^, 161.0230 [M−H−C_9_H_6_O_3_−C_7_H_12_O_6_]^−^, 173.0423 [M−H−2C_9_H_6_O_3_−H_2_O]^−^, 155.0321 [M−H−2C_9_H_6_O_3_−2H_2_O]^−^	Isochlorogenic acid A
	25.681	C_25_H_24_O_12_	515.1178	353.0851 [M−H−C_9_H_6_O_3_]^−^, 173.0429 [M−H−2C_9_H_6_O_3_−H_2_O]^−^, 179.0324 [M−H−C_9_H_6_O_3_−C_7_H_10_O_5_]^−^, 191.0527 [M−H−2C_9_H_6_O_3_]^−^, 135.0431 [M−H−C_9_H_6_O_3_−C_7_H_10_O_5_−CO_2_]^−^, 155.0336 [M−H−2C_9_H_6_O_3_−2H_2_O]^−^	173.0425 [M−H−2C_9_H_6_O_3_−H_2_O]^−^, 179.0325 [M−H−C_9_H_6_O_3_−C_7_H_10_O_5_]^−^, 135.0429 [M−H−C_9_H_6_O_3_−C_7_H_10_O_5_−CO_2_]^−^, 191.0533 [M−H−2C_9_H_6_O_3_]^−^, 155.0325 [M−H−2C_9_H_6_O_3_−2H_2_O]^−^, 161.0210 [M−H−C_9_H_6_O_3_−C_7_H_12_O_6_]^−^	Isochlorogenic acid C

For monocaffeoylquinic acids, in negative ion mode, if CGA (C_16_H_17_O_9_, *m/z* 353) loses one molecule of caffeoyl group (C_9_H_6_O_3_, *m/z* 162), it yields quinic acid (C_7_H_11_O_6_, *m/z* 191). In negative ion mode, the caffeoyl group (C_9_H_6_O_3_, *m/z* 162) loses a proton to form a fragment ion with *m/z* 161. When CGA (C_16_H_17_O_9_, *m/z* 353) loses one molecule of caffeic acid (C_9_H_7_O_4_, *m/z* 179), the remaining moiety yields a fragment ion with *m/z* 174. Quinic acid (C_7_H_11_O_6_, *m/z* 191) loses one molecule of water to form dehydrated quinic acid fragment at *m/z* 173; on this basis, if it loses another molecule of water, it yields a fragment ion with *m/z* 155, and if it undergoes decarboxylation upon heating, it forms a fragment ion with *m/z* 127. The caffeoyl group (C_9_H_6_O_3_, *m/z* 162) loses one molecule of water to yield a fragment ion with *m/z* 143. After caffeic acid (C_9_H_7_O_4_, *m/z* 179) undergoes decarboxylation upon heating, it forms a fragment ion with *m/z* 135.

For dicaffeoylquinic acids, in negative ion mode, isochlorogenic acid (C_25_H_24_O_12_, *m/z* 515) loses one molecule of caffeoyl group (C_9_H_6_O_3_, *m/z* 162) to yield monocaffeoylquinic acids (*m/z* 353), which then lose one molecule of water to form dehydrated monocaffeoylquinic acids at *m/z* 335. Most of the other fragment ion peaks are obtained based on the fragmentation of monocaffeoylquinic acids. The ion abundance spectra of above‐mentioned MS data for the six compounds is included in Supporting Information 2.

### 3.2. The Thermal Stability of the CGAs in *LjF*

#### 3.2.1. The Thermal Stability of the Monocaffeoylquinic Acids

**Figure 4:**
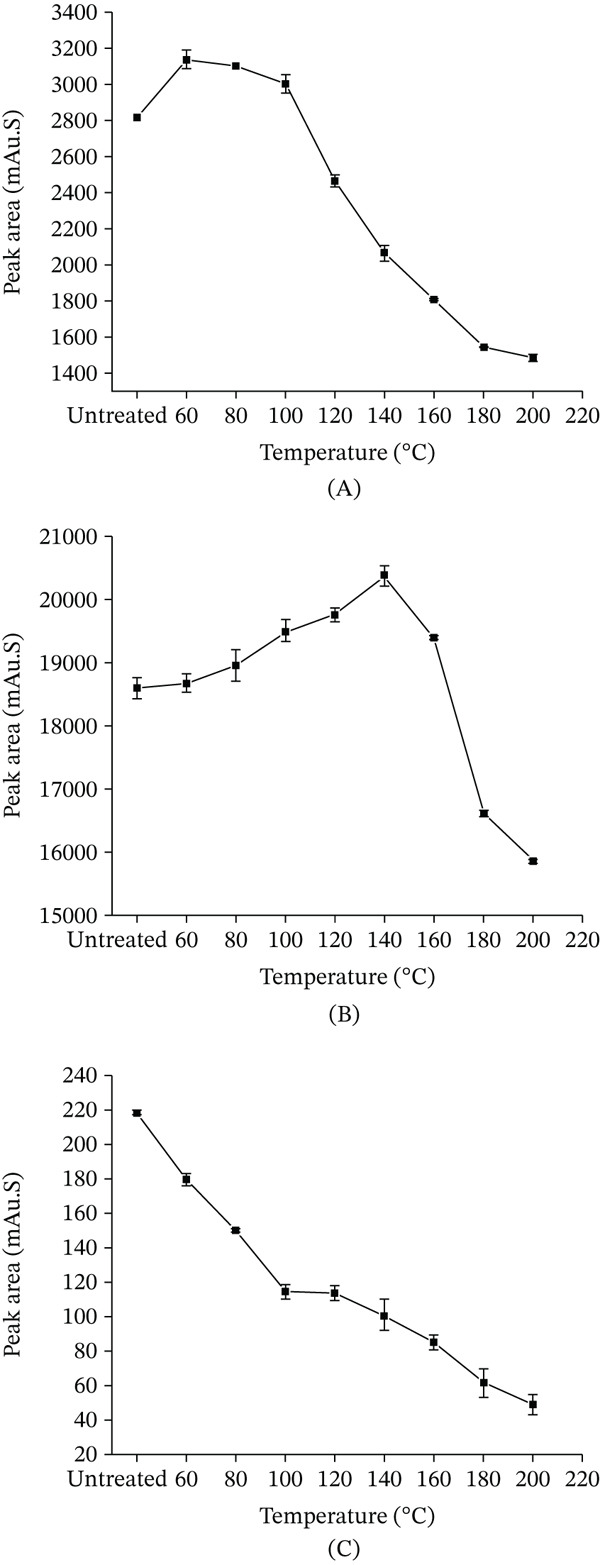
Effect of heating temperature on the peak areas of different CGAs (15‐min treatment). (A) neochlorogenic acid; (B) chlorogenic acid; and (C) cryptochlorogenic acid. Data are presented as mean ± RSD (*n* = 3).

As illustrated in Figure 4A, the neochlorogenic acid increased relative to the untreated *LjF* sample following heat treatment at 60°C, 80°C, and 100°C. Subsequently, a progressive decline was observed with further temperature elevation. Neochlorogenic acid concentration exhibits an initial increase followed by a decrease postheat treatment. This pattern indicates generation of new neochlorogenic acid within a specific temperature range; however, at higher temperatures, its reduction exceeded generation. Notably, a sharp decline occurred between 100°C and 180°C. Compared with the untreated sample, the neochlorogenic acid was elevated at 60°C, whereas under the destructive condition of 200°C, it was reduced.

As depicted in Figure 4B, the CGA progressively increased following heat treatment within the temperature range of 60°C–140°C, achieving a maximum enhancement relative to the untreated sample. Beyond 140°C, the content underwent a rapid decline, decreasing to 22.20% of its peak value and 14.73% of the level observed in the untreated sample. This behavior demonstrates that CGA, analogous to neochlorogenic acid, exhibits an initial concentration increase followed by a decrease upon more extreme thermal processing. This signifies generation of new CGA within a specific thermal range, succeeded by degradation processes exceeding its generation rate at elevated temperatures.

As shown in Figure 4C, the cryptochlorogenic acid decreased significantly following heat treatment. This indicates that the degradation and transformation of cryptochlorogenic acid under thermal conditions exceeded its rate of generation.

**Figure 5:**
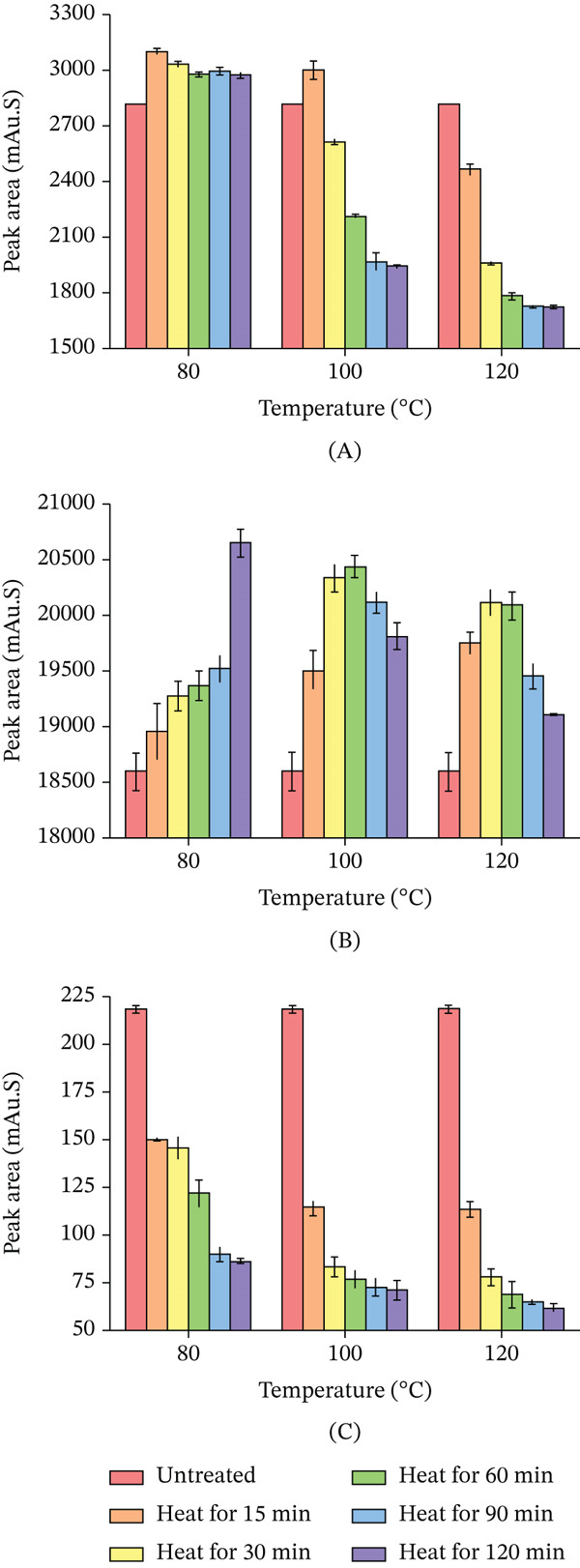
Changes in the peak area of CGAs under various heat treatment conditions. (A) neochlorogenic acid; (B) chlorogenic acid; and (C) cryptochlorogenic acid. Data are presented as mean ± RSD (*n* = 3).

Figure 5A demonstrates that neochlorogenic acid was elevated relative to the untreated sample after heating at 80°C. Though this elevation diminished with prolonged heating at 80°C, it indicates that the reduction lagged behind the generation. At 100°C, neochlorogenic acid increased after 15 min of heating and subsequently declined; the rate of decline decelerated after 90 min. It signifies that initially, production exceeded reduction; as time elapses, reduction surpasses production, with eventual equilibrium. At 120°C, neochlorogenic acid underwent a substantial immediate decrease, with the declining trend slowing later; this reflects accelerated initial degradation at higher temperatures, ultimately reaching a quasi‐equilibrium. For identical durations, higher temperatures were observed to reduce its content, similar to Figure 4A.

Figure 5B illustrates CGA after heat treatment at 80°C exceeded the untreated *LjF* sample. It increased with extended heating duration and reached maximum concentration at 120 min. At 100°C, CGA increased during the initial 15–60 min and remained higher than the untreated sample. A turning point emerged at 60 min, after which it declined from the peak yet still exceeded the untreated sample. At 120°C, CGA similarly rose initially then reduced with an earlier inflection point at 30 min compared with 60 min at 100°C, and all remained higher than the untreated sample. With 15 min of heating, CGA rose with increasing temperature; whereas for 120 min, it decreased as temperature increased. After heating for 30, 60, and 90 min, it first increased then decreased, reflecting dynamic shifts in relative rates of generation and degradation across the temperature gradient. Figure 5C demonstrates that under 80°C heating for 90 min, the cryptochlorogenic acid decreased, whereas from 90 to 120 min, it underwent a further reduction. At 100°C, heating for 30 min, cryptochlorogenic acid plunged, whereas a slow reduction occurring between 30 and 120 min. Under 120°C conditions, the content declined more sharply in the period immediately after 30 min of heating than it did from 30 to 120 min. The cryptochlorogenic acid consistently decreased with prolonged heating time, with the reduction rate gradually slowing until relative equilibrium was reached. Furthermore, elevated temperatures not only enhanced the initial rate of decline but also induced a reduction in the final equilibrium content attained following prolonged heating with increasing temperature.

#### 3.2.2. The Thermal Stability of the Dicaffeoylquinic Acids

**Figure 6:**
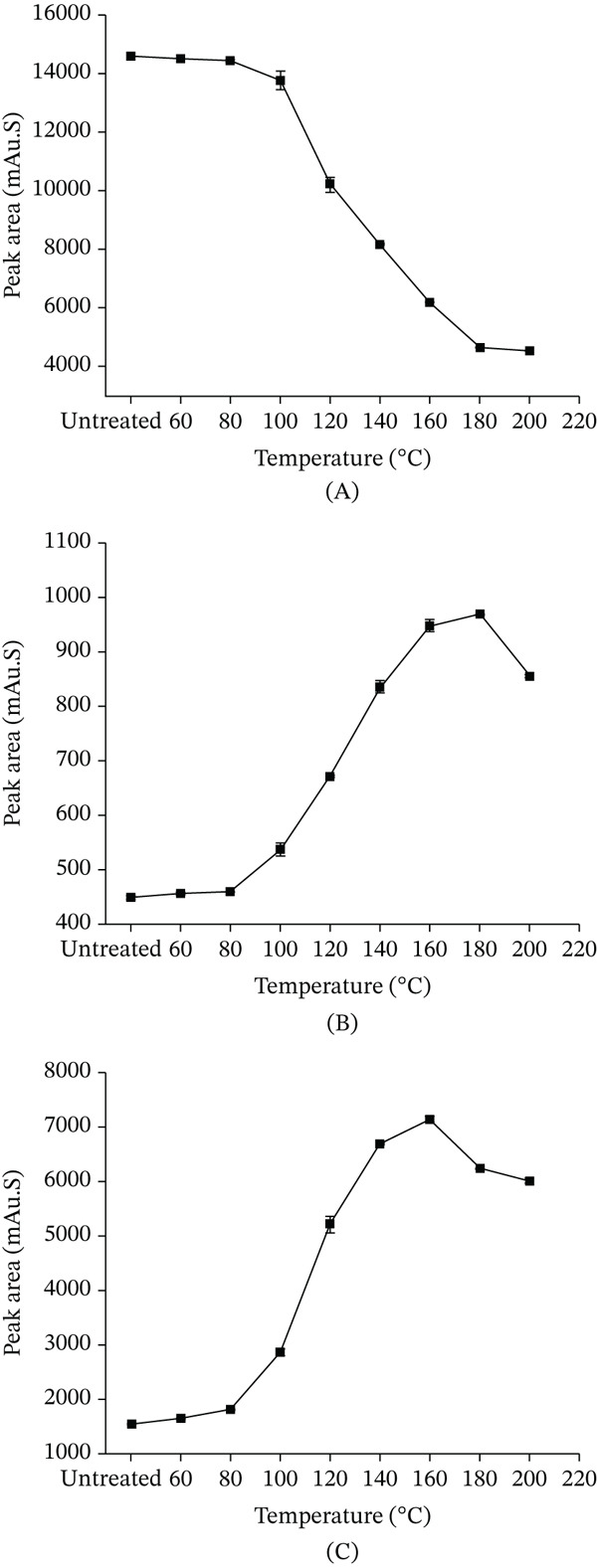
Effect of heating temperature on the peak area of different CGAs (15‐min treatment). (A) isochlorogenic acid A; (B) isochlorogenic acid B; and (C) isochlorogenic acid C. Data are presented as mean ± RSD (*n* = 3).

As shown in Figure 6A, the isochlorogenic acid A decreased significantly following heat treatment. Within the temperature range of 60°C–100°C, the extent of its decomposition and transformation was relatively limited. However, a substantial increase in decomposition and transformation was observed between 100°C and 180°C. Beyond this temperature range, the content stabilized. These results indicate that under thermal conditions, the degradation and transformation of isochlorogenic acid A exceeded its generation. As shown in Figure 6B, isochlorogenic acid B gradually increased with heat treatment at 60°C–180°C: slow increase at 60°C–80°C, accelerating notably at 80°C–180°C, peaking at 180°C versus the untreated sample. Beyond 180°C, it decreased. This pattern, similar to CGA and neochlorogenic acid, shows that initial increase then decrease within a specific temperature range, indicating generation in a thermal window followed by degradation and transformation dominance at higher temperatures. As shown in Figure 6C, isochlorogenic acid C increased gradually with heat treatment at 60°C–160°C, with a marked increase at 80°C–140°C that was faster than that at 60°C–80°C and 140°C–160°C. It peaked at 160°C, significantly higher than the untreated sample, and declined beyond 160°C. Like CGA, neochlorogenic acid, and isochlorogenic acid B, it first increased then decreased upon thermal processing, indicating generation in a specific range followed by degradation dominance at elevated temperatures. Its inflection point was 20°C earlier than that of isochlorogenic acid B.

**Figure 7:**
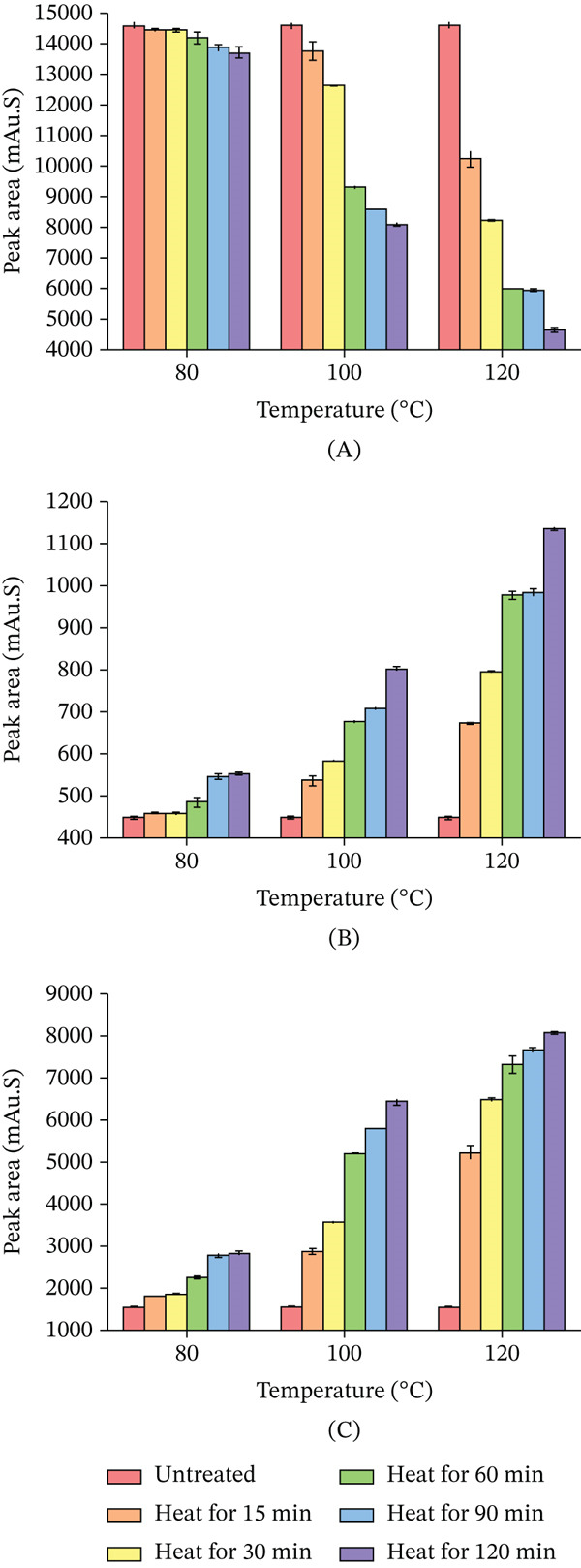
Changes in the peak areas of CGAs under various heat treatment conditions. (A) isochlorogenic acid A; (B) isochlorogenic acid B; and (C) isochlorogenic acid C. Data are presented as mean ± RSD (*n* = 3).

Figure 7A illustrates that isochlorogenic acid A decreased at 80°C after 120 min of heat treatment, with greater reductions at 100°C and 120°C, in increasing order. It consistently declined with prolonged heating time. Higher temperatures accelerated the decrease rate and resulted in a greater overall reduction magnitude. Figure 7B demonstrates that after 120 min of heat treatment at 80°C, 100°C, and 120°C, the isochlorogenic acid B increased, with the increase becoming more pronounced as temperature rose. It consistently increased with prolonged heating across all tested temperatures. Higher temperatures enhanced both the magnitude and rate of increase, leading to greater elevation at equivalent durations. Data suggest temperature elevation influences its increase more significantly than extended heating time.

Figure 7C demonstrates that isochlorogenic acid C exhibited a content change pattern similar to isochlorogenic acid B. After heating for 120 min, it increased, with the increment more marked at higher temperatures. Furthermore, the variation of isochlorogenic acid C exhibited greater sensitivity to temperature changes compared with isochlorogenic acid B.

#### 3.2.3. Analysis of Component Changes

Principal component analysis (PCA) was employed to evaluate the combined effects of temperature and time on the compositional changes of CGAs in *LjF*. By integrating score and loading plots, the study is aimed at identifying key chemical markers and optimal processing conditions that maximize sample discriminability.

**Figure 8:**
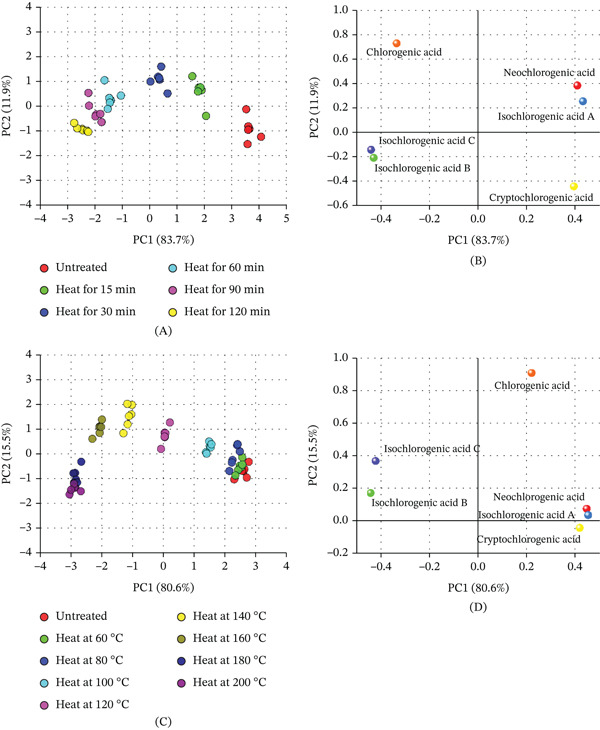
PCA plots of chlorogenic acids in *LjF* under different thermal processing conditions. (A) PCA score plot for samples heated at 100°C for different durations (15–120 min); (B) PCA loading plot under heat treatment at 100°C; (C) PCA score plot for samples heated at different temperatures (60°C–200°C) for 15 min; and (D) PCA loading plot under 15 min of heat treatment.

In Figure 8A, when examining the effect of heating time at 100°C, PCA demonstrated that most compositional changes occurred within the first 30 min. Samples treated for 60–120 min clustered closely, suggesting limited further transformation. In Figure 8B, the PC1 axis was mainly influenced by isochlorogenic acids A and C, whereas PC2 was driven by CGA and, to a lesser extent, cryptochlorogenic acid. The convergence of scores over time indicated reaction stabilization, with extended heating offering no additional discriminatory benefit.

In Figure 8C, under 15 min of heat treatment, PCA revealed a clear temperature‐dependent segregation of samples. The 100°C–160°C range exhibited the greatest dispersion along PC1, indicating the most significant chemical differentiation. In Figure 8D, loading analysis showed that isochlorogenic acid A (positive loading) and isochlorogenic acids B and C (negative loadings) were the primary contributors to this separation. PC2 was dominated by CGA, whose trend of initial increase followed by decrease aligned with the observed score pattern, reflecting complex degradation and interconversion dynamics.

In conclusion, PCA results effectively distinguishes *LjF* samples based on CGA profiles under thermal treatment. Temperature is the dominant factor, with 100°C–160°C being the most informative range, whereas time effects plateau after 30 min. The six CGAs, particularly dicaffeoylquinic acids and CGA, serve as reliable chemical markers for monitoring thermal processing, providing a basis for quality control and optimization in functional food production.

#### 3.2.4. The Degradation and Transformation Pathway of Isochlorogenic Acid A

Based on mass spectrometric analysis of isochlorogenic acid A, it is proposed that the kinetic and thermodynamic degradation products of isochlorogenic acid A are distinct. This finding contrasts with reports in the literature [32, 33], which contend that CGA is its sole degradation product. We designed the experiment described in Section 2.2.4 to verify the hypothesis.

**Figure 9:**
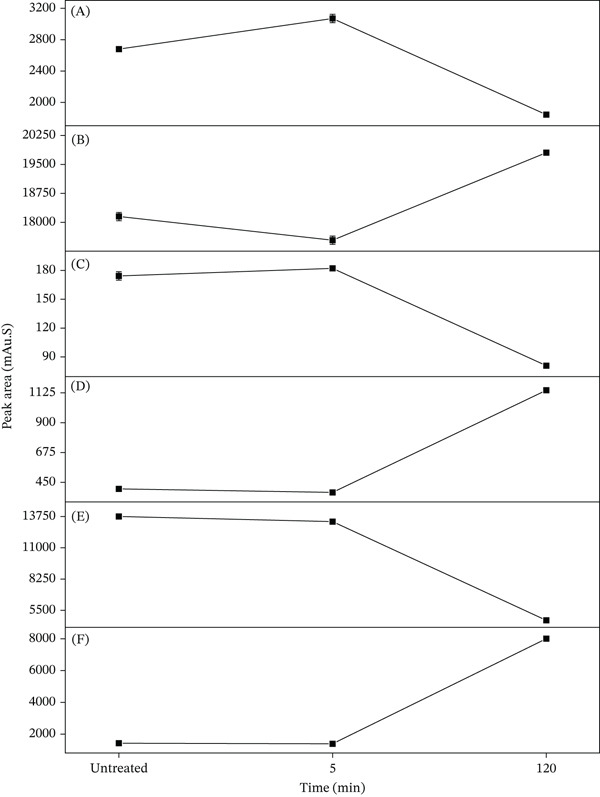
Relationship between heat treatment time and the peak areas. (A) neochlorogenic acid; (B) chlorogenic acid; (C) cryptochlorogenic acid; (D) isochlorogenic acid B; (E) isochlorogenic acid A; and (F) isochlorogenic acid C. Data are presented as mean ± RSD (*n* = 3).

As shown in Figure 9, after treatment at 120°C for 5 min, the content of neochlorogenic acid increased significantly by 15.05%, whereas that of CGA decreased by 3.41%. The concentration of cryptochlorogenic acid rose by 4.02%, whereas isochlorogenic acids B, A, and C decreased by 5.30%, 3.73%, and 2.07%, respectively. The decrease in isochlorogenic acid A after short‐term high‐temperature treatment indicates its transformation or degradation to other compounds. Moreover, isochlorogenic acids B and C also decreased slightly, indicating that isochlorogenic acid A tends to lose one caffeoyl group to form monocaffeoylquinic acids during thermal transformation, rather than generating other dicaffeoylquinic acids via positional isomerization of caffeoyl moieties. Upon further heating at 120°C for 120 min, neochlorogenic acid declined and CGA increased. Cryptochlorogenic acid was drastically reduced by 53.97% compared with the untreated sample, and isochlorogenic acids B and C were remarkably elevated by 184.8% and 463.27%, respectively, whereas isochlorogenic acid A exhibited a decreasing trend.

**Figure 10:**
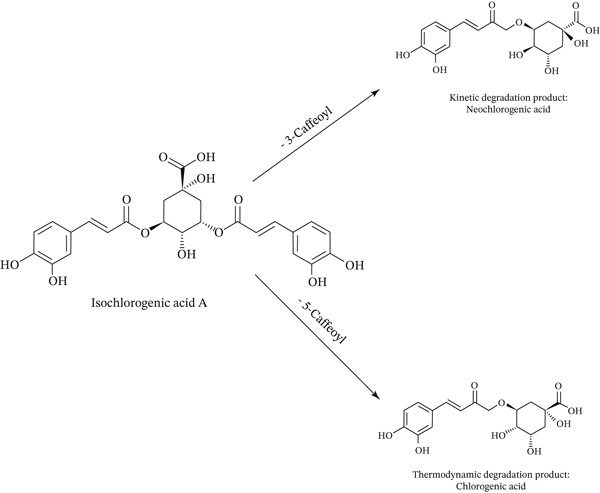
The degradation pathway of isochlorogenic acid A.

As depicted in Figure 1, the two caffeic acid units in isochlorogenic acid A are esterified at the 3‐ and 5‐hydroxyl groups of the quinic acid core. As illustrated in Figure 10, while cleavage at the 5‐position produces CGA, cleavage of the ester bond at the 3‐position yields neochlorogenic acid. CGA is thermodynamically favored under prolonged heating, whereas neochlorogenic acid tends to form as a transient intermediate. Specifically, during extended heating periods, this class of compounds exhibits a preferential transformation toward CGA rather than neochlorogenic acid. Namely, the observed content changes—increased neochlorogenic acid and decreased CGA following brief high‐temperature exposure—indicate that the rise in neochlorogenic acid primarily originates from thermal degradation of isochlorogenic acid A rather than interconversion among other monocaffeoylquinic acids. Collectively, these findings demonstrate that isochlorogenic acid A preferentially generates neochlorogenic acid under short‐term high‐temperature conditions, leading us to conclude that neochlorogenic acid serves as its kinetic degradation product.

Is the thermodynamic degradation product of isochlorogenic acid A also neochlorogenic acid? Likewise, how to explain the phenomenon of increased CGA caused by the thermal degradation of isochlorogenic acid A? The neochlorogenic acid increases significantly after short‐term heat treatment but decreases markedly following prolonged exposure. Conversely, CGA decreases initially after heating but increases after extended treatment. This differential behavior indicates shifting generation and degradation rates between these two compounds with prolonged heating duration. More specifically, it is concluded that the primary degradation products of isochlorogenic acid A shift from neochlorogenic acid under kinetic control to CGA under thermodynamic control. Consequently, it is demonstrated that the degradation products of isochlorogenic acid A consist of neochlorogenic acid as the kinetic product under short‐term conditions and CGA as the thermodynamic product following extended thermal exposure.

According to the reported work [20], the direct synthesis of diacyl compounds, without enzyme catalysis, from monocaffeoylquinic acids under mild heating is challenging, requiring more stringent conditions to form dicaffeoylquinic acids. Research work [34] also reports that in caffeoylquinic acids, caffeic acid can migrate to different binding sites on the quinic acid core under heating, namely, intramolecular acyl transfer occurs. Therefore, the increase in isochlorogenic acid B (3,4‐dicaffeoylquinic acid) and isochlorogenic acid C (4,5‐dicaffeoylquinic acid) may also primarily stem from isochlorogenic acid A (3,5‐dicaffeoylquinic acid).

In summary, isochlorogenic acid A not only decomposes into its kinetic degradation product, neochlorogenic acid, and its thermodynamic degradation product, CGA, under different heating conditions, but also undergoes intramolecular acyl migration, where one caffeic acid moiety migrates to a different hydroxyl group on the quinic acid, to form isochlorogenic acids B and C.

### 3.3. The Transformation of CGAs After Heat Treatment

**Figure 11:**
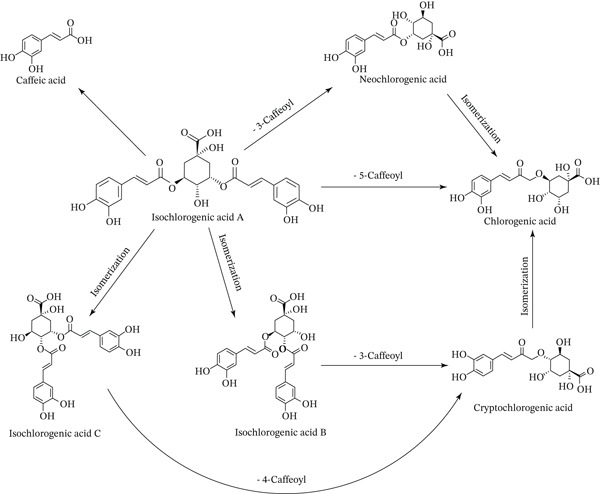
The pathways for the conversion among CGAs.

Based on the descriptions and preliminary analyses in Sections 3.2.1–3.2.4, the result clearly demonstrate that isochlorogenic acid A, cryptochlorogenic acid, and neochlorogenic acid decreased significantly after heat treatment, whereas CGA, isochlorogenic acid B, and isochlorogenic acid C increased. Through analysis of the structures of these six compounds, relevant works [34–36], and experimental data, the dynamic transformation patterns of six key CGAs under dry‐heating treatment were systematically elucidated. Figure 11 illustrates the proposed interconversion pathways among the six major CGAs, centered around isochlorogenic acid A.

In light of the analysis in Section 3.2.4, it is proposed that isochlorogenic acid A is converted into isochlorogenic acids B and C upon heat exposure. Notably, after heat treatment, among the three dicaffeoylquinic acid isomers, the increase of isochlorogenic acid C is greater than that of isochlorogenic acid B. This suggests that under the provided dry‐heat conditions, isochlorogenic acid C is likely the thermodynamically most stable configuration. It is speculated that this may be attributed to the more symmetrical arrangement of the two caffeoyl groups in isochlorogenic acid C, which results in a more stable overall molecular conformation.

Structural and mass spectrometric analysis of three dicaffeoylquinic acids indicates that dicaffeoylquinic acids primarily lose one caffeoyl moiety under thermal stress, generating monocaffeoylquinic acids. Two potential cleavage sites exist between the caffeic acid and quinic acid units in every dicaffeoylquinic acid. Via mass spectrometric analysis, high similarity in spectral characteristics between the spectra of isochlorogenic acid B (3,4‐dicaffeoylquinic acid) and isochlorogenic acid C (4,5‐dicaffeoylquinic acid) was revealed. Meanwhile, their secondary mass spectra also showed significant similarity to that of cryptochlorogenic acid (4‐O‐caffeoylquinic acid). It is therefore inferred that during mass spectrometric fragmentation, the 3‐position acyl group in isochlorogenic acid B and the 5‐position acyl group in isochlorogenic acid C are preferentially cleaved, yielding characteristic fragments of cryptochlorogenic acid. This indicates that under the experimental condition of short‐term heating, the main degradation product of both isochlorogenic acid B and isochlorogenic acid C is cryptochlorogenic acid. Although cryptochlorogenic acid was formed from isochlorogenic acid B and C under short‐term heating, its content still declined as the heat treatment time extended.

The decrease of cryptochlorogenic acid and neochlorogenic acid as well as the increase of CGA were observed. These three monocaffeoylquinic acids undergo mutual conversion during heating; however, under elevated temperatures, both cryptochlorogenic acid and neochlorogenic acid tend to convert into CGA as CGA constitutes the thermodynamically stable component among the three monocaffeoylquinic acids. Furthermore, all monocaffeoylquinic acids also undergo further degradation. This explains the decrease of cryptochlorogenic acid and neochlorogenic acid and the increase of CGA.

Upon high‐temperature heating of *LjF*, three main types of interconversions occur among its six major CGAs. The first involves mutual transformations within monocaffeoylquinic acids, ultimately stabilizing as CGA. The second entails interconversions within dicaffeoylquinic acids; under the experimental conditions employed in this study, isochlorogenic acid A transforms into isochlorogenic acids B and C, with the latter being the more stable thermodynamic product. The third type is the conversion of dicaffeoylquinic acids to monocaffeoylquinic acids.

## 4. Conclusion

In conclusion, this work elucidates the thermal degradation and transformation of CGAs in *LjF* under dry heating. We demonstrate that dry heating drives distinct conversion pathways: the interconversion of neochlorogenic and cryptochlorogenic acids to CGA, and the isomerization of isochlorogenic acid A to B/C via intramolecular acyl migration, concurrently yielding neochlorogenic acid (kinetically favored) and CGA (thermodynamically stable). These findings establish the first comprehensive thermal reaction network for CGAs in *LjF*, providing a scientific foundation for optimizing processing parameters to enhance the quality and efficacy of *LjF*‐derived products.

## Funding

This study was supported by National Natural Science Foundation of China (Nos. 81573397 and 81973285).

## Conflicts of Interest

The authors declare no conflicts of interest.

## Supporting Information

Additional supporting information can be found online in the Supporting Information section.

## Supporting information


**Supporting Information 1** The method validation of the high‐performance liquid chromatography (HPLC) adopted in this study, covering accuracy, stability, repeatability, and recovery.

**Supporting Information 2** The mass spectrometry (MS) data of ion abundance for the six compounds.

## Data Availability

The data that support the findings of this study are available from the corresponding authors upon reasonable request.
