# Supplementary material for: Investigating the Thermal Transformations of Chlorogenic Acids During Dry‐Heating Processing of Lonicerae Japonicae Flos
Source: Int J Food Sci. 2026 May 11;2026:7566064. doi: 10.1155/ijfo/7566064 (PMC13159092; doi:10.1155/ijfo/7566064)
Supplement: Supplementary file 1 — Supporting Information 1 The method validation of the high‐performance liquid chromatography (HPLC) adopted in this study, covering accuracy, stability, repeatability, and recovery. [file IJFO-2026-7566064-s001.docx]

**Supporting Information 1**

**HPLC Method Validation**

**1. Precision**

A precisely weighed 100.0 mg of untreated *Lonicerae japonicae Flos* (*LjF*) sample was immersed in 10 mL of 80% (V/V) methanol and subjected to ultrasonic extraction for 60 min (power: 300 W, frequency: 40 kHz). After filtration, the solution served as the sample for precision testing. Intra-day precision was assessed by performing three consecutive injections per day under the same HPLC conditions detailed in Section 2.2.2, while inter-day precision was evaluated by repeating this analysis over three consecutive days. The intra-day precision RSD values for neochlorogenic acid, chlorogenic acid, cryptochlorogenic acid, isochlorogenic acid A, isochlorogenic acid B, and isochlorogenic acid C were 0.38%, 0.38%, 0.20%, 0.33%, 0.43%, and 0.18%, respectively. Their inter-day precision RSD values were 1.88%, 0.76%, 0.71%, 1.28%, 1.00%, and 1.49%, respectively. These results demonstrate excellent instrument precision.

**2. Stability**

To assess stability, seven aliquots of untreated *LjF* sample (100.0 mg each) were accurately weighed and individually extracted with 10 mL of 80% (v/v) methanol *via* ultrasonication for 60 min (power: 300 W, frequency: 40 kHz). After filtration, the extracts were stored at 4°C and analyzed under the HPLC conditions described in Section 2.2.2 at 0, 1, 2, 4, 6, 12, and 24 hours. The RSD values for the stability of neochlorogenic acid, chlorogenic acid, cryptochlorogenic acid, isochlorogenic acid A, isochlorogenic acid B, and isochlorogenic acid C were 3.44%, 1.87%, 2.36%, 1.70%, 1.78%, and 2.75%, respectively, demonstrating good sample stability.

**3. Repeatability**

Repeatability was evaluated using six independent aliquots of untreated *LjF* sample (100.0 mg each). Each was extracted in 10 mL of 80% (V/V) methanol via 60-min ultrasonication (power: 300 W, frequency: 40 kHz) and filtered. All extracts were analyzed under the same HPLC conditions (Section 2.2.2). The repeatability RSD values for the six chlorogenic acids (neochlorogenic acid, chlorogenic acid, cryptochlorogenic acid, isochlorogenic acid A, isochlorogenic acid B, and isochlorogenic acid C) were 1.66%, 1.06%, 1.99%, 1.32%, 1.60%, and 1.85%, respectively, confirming excellent method repeatability.

**4. Recovery**

For recovery testing, six aliquots of untreated *LjF* sample (100.0 mg each) were individually extracted in 10 mL of 80% (V/V) methanol via ultrasonication for 60 min (power: 300 W, frequency: 40 kHz) and filtered. A 0.5 mL portion of each filtrate was mixed with 0.1 mL of the reference standard solution. The mixture was then diluted to 1.0 mL with 80% (V/V) methanol and vortexed thoroughly to form the recovery test sample. Analysis was performed under the HPLC conditions described in Section 2.2.2. The average recovery rates for chlorogenic acid, isochlorogenic acid A, isochlorogenic acid B, and isochlorogenic acid C were 101.7%, 101.2%, 103.2%, and 99.2%, respectively, with corresponding RSD values of 3.5%, 2.3%, 2.6%, and 2.4% across the six replicates. Based on these results, the established method demonstrates satisfactory recovery performance.
