# Supplementary material for: Investigating the Thermal Transformations of Chlorogenic Acids During Dry‐Heating Processing of Lonicerae Japonicae Flos
Source: Int J Food Sci. 2026 May 11;2026:7566064. doi: 10.1155/ijfo/7566064 (PMC13159092; doi:10.1155/ijfo/7566064)
Supplement: Supplementary file 2 — Supporting Information 2 The mass spectrometry (MS) data of ion abundance for the six compounds. [file IJFO-2026-7566064-s002.docx]

**Supporting Information 2**

**Ion Abundance Spectra of CGAs Under MS/MS**

Figure S1. Neochlorogenic acid (m/z=353.0854) product ion abundance spectrum under MS/MS at 20 eV.

Figure S2 Neochlorogenic acid (m/z=353.0854) product ion abundance spectrum under MS/MS at 40 eV.

Figure S3. Chlorogenic acid (m/z=353.0854) product ion abundance spectrum under MS/MS at 20 eV.

Figure S4. Chlorogenic acid (m/z=353.0854) product ion abundance spectrum under MS/MS at 40 eV.

Figure S5. Cryptochlorogenic acid (m/z=353.0854) product ion abundance spectrum under MS/MS at 20 eV.

Figure S6. Cryptochlorogenic acid (m/z=353.0854) product ion abundance spectrum under MS/MS at 40 eV.

Figure S7. Isochlorogenic acid B (m/z=515.1174) product ion abundance spectrum under MS/MS at 20 eV.

Figure S8. Isochlorogenic acid B (m/z=515.1174) product ion abundance spectrum under MS/MS at 40 eV.

Figure S9. Isochlorogenic acid A (m/z=515.1174) product ion abundance spectrum under MS/MS at 20 eV.

Figure S10. Isochlorogenic acid A (m/z=515.1174) product ion abundance spectrum under MS/MS at 40 eV.

Figure S11. Isochlorogenic acid C (m/z=515.1174) product ion abundance spectrum under MS/MS at 20 eV.

Figure S12. Isochlorogenic acid C (m/z=515.1174) product ion abundance spectrum under MS/MS at 40 eV.
